# Effects of elastic resistance exercise on body composition and physical capacity in older women with sarcopenic obesity: A CONSORT-compliant prospective randomized controlled trial

**DOI:** 10.1097/MD.0000000000007115

**Published:** 2017-06-08

**Authors:** Chun-De Liao, Jau-Yih Tsauo, Li-Fong Lin, Shih-Wei Huang, Jan-Wen Ku, Lin-Chuan Chou, Tsan-Hon Liou

**Affiliations:** a School and Graduate Institute of Physical Therapy, College of Medicine, National Taiwan University; b Department of Physical Medicine and Rehabilitation; c Department of Radiology, Shuang Ho Hospital; d Department of Physical Medicine and Rehabilitation, School of Medicine, College of Medicine; e Graduate Institute of Injury Prevention and Control, Taipei Medical University, Taipei, Taiwan.

**Keywords:** body composition, elastic resistance training, muscle quality, physical mobility, sarcopenic obesity

## Abstract

**Background:**

Sarcopenia is associated with loss of muscle mass and an increased risk of physical disability in elderly people. However, the prevalence of sarcopenia has increased in obese elderly populations. The purpose of this study was to identify the clinical efficacy of elastic resistance exercise training (RET) in patients with sarcopenic obesity.

**Methods:**

This study was conducted at the rehabilitation center of a university-based teaching hospital and was designed as a prospective and randomized controlled trial with an intention-to-treat analysis. A total of 46 women aged 67.3 (5.2) years were randomly assigned to an experimental group (EG) and control group (CG). The EG underwent elastic RET for 12 weeks, and the CG received no RET intervention. All outcome measures were assessed at the baseline and posttest, including body composition measured using dual-energy X-ray absorptiometry, muscle quality (MQ) defined as a ratio of muscular strength to muscle mass, and physical capacity assessed using functional mobility tests. One-way analysis of covariance and Pearson correlation were used to compare outcomes between the 2 groups and to identify the relationship between changes in body composition and physical outcomes, respectively. A chi-square test was performed to identify differences in qualitative data between the 2 groups.

**Results:**

At the posttest, a significant between-group difference was observed in fat-free mass, MQ, and physical capacity (all *P* < .05); and a significant correlation was found between leg-lean-mass change and gait speed (*r* = 0.36; *P* < .05). After 12 weeks of elastic RET intervention, the EG had significantly fewer patients exhibiting sarcopenia (*P* < .05) and experiencing physical difficulty (*P* < .001) than the CG.

**Conclusion:**

The present data suggest that elastic resistance exercise exerted benefits on the body composition, MQ, and physical function in patients with sarcopenic obesity. Regular exercise incorporating elastic RET should be used to attenuate muscle mass loss and prevent physical difficulty in obese older adults with sarcopenia on reconditioning therapy.

Trial Registration: Chinese Clinical Trial Registry, ChiCTR-IPR-15006069.

## Abstract 2

Supplemental Digital Content is available in the text

## 1 Introduction

Sarcopenic obesity, a recently identified phenotype in obese elderly populations, is an additive effect of sarcopenia and obesity. Sarcopenia is termed and characterized by age-related muscle atrophy.^[1]^ Obesity, resulting from an increase in adipose tissue, is considered a critical cause of skeletal muscle loss that leads to a cycle of continuous fat gain.^[2]^ Sarcopenia or obesity itself is independently associated with physical disability in elderly people,^[3–5]^ whereas sarcopenic obesity results in more physical limitations than sarcopenia or obesity alone.^[6–10]^

Aging-related loss of skeletal muscle mass, primarily characterized by type II myofiber phenotype atrophy, has been revealed to account for a smaller muscle fiber size rather than the loss in fiber number.^[11,12]^ Studies have reported that resistance exercise training (RET) alleviated aging-related type II myofiber phenotype atrophy through satellite cell proliferation and an increase in the rate of muscle contractile and mitochondrial protein synthesis, which further contributed to myofiber hypertrophy.^[12,13]^

Resistance-type training involving elastic bands has been frequently used as a treatment method and is considered safe for muscle strengthening in elderly people.^[14,15]^ Muscle activations in and self-perceived efficacy of this training are similar to those of free-weight resistance training.^[16]^ Elastic RET is not only safe for elderly people^[15]^ but also improves muscle quality (MQ),^[17]^ which is determined as a ratio of muscle strength or power relative to muscle mass.^[18]^

Previous studies have supported the benefits of elastic resistance exercise by using Theraband elastic strips or tubing for preventing either obesity or sarcopenia.^[14,19–21]^ However, the effects of elastic RET on body composition, MQ, and physical function in elderly women with sarcopenic obesity remain unclear. The purpose of the present study was to identify the effects of an elastic resistance exercise regime on the body composition, MQ, and physical capacity of elderly women with sarcopenic obesity and to determine the association between changes in body composition and mobility outcome after exercise intervention. Furthermore, we examined whether RET reduced the risk of physical difficulty.

## 2 Methods

### 2.1 Study design

The present clinical trial involved an experimental design, and the study protocol was executed at the rehabilitation center of Shuang Ho Hospital, Taipei Medical University. Both patients and examiners were blinded to the group assignment. All patients were enrolled from April 2015 to January 2016. The patients provided informed consent and were then randomized into 2 groups: an experimental group (EG), receiving elastic resistance training, and an age-matched control group (CG). A standard medical chart review for each included patient was performed to assess the prevalent comorbidities, and the comorbidity scores were calculated using the Cumulative Illness Rating Scale (CIRS).^[22]^ All outcome measure data were collected at the baseline (pretest) and after 12 weeks of exercise intervention (posttest). This study was approved by the Joint Institutional Review Board of Taipei Medical University (Trial number: 201306019) and registered at the Chinese Clinical Trial Registry (Registry number: ChiCTR-IPR-15006069).

### 2.2 Study population

**Figure 1:**
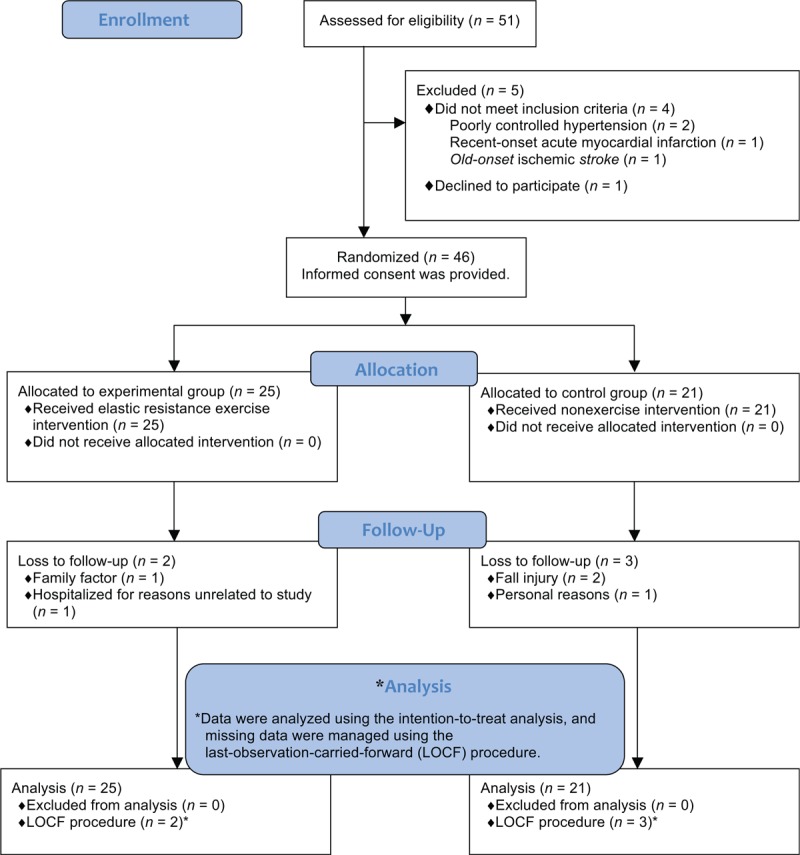
CONSORT flow diagram of patient selection and allocation.

Eligible female patients were enrolled on the basis of the CONSORT Statement Extension for Randomized Controlled Trials of Nonpharmacological Trials, as displayed in the flowchart in Fig. 1.^[23]^ Eligible female patients aged 60 to 80 years were correlatively selected and recruited from the outpatient department of a rehabilitation center in our hospital. Before recruitment, each patient was screened for eligibility and whether she met the criteria for obesity and sarcopenia. Eligible patients who were defined as having sarcopenic obesity were excluded if they had any of the following conditions: poorly controlled hypertension by use of medications such as antihypertensive medication; any joint contracture or internal metal implant, such as total joint arthroplasty; any cardiovascular disease, such as unstable angina, recent acute myocardial infarction, and heart failure, and any pulmonary illness, such as chronic obstructive pulmonary disease, that would prevent them from engaging in an exercise study; or neurological impairments or disorders, such as cerebral vascular accident and Parkinson disease, that impaired mobility.

Because research has identified significant sex differences in response to RET in older people,^[24]^ and because the criteria for identifying sarcopenia differ between men and women,^[25,26]^ merging the collected data of the 2 sexes into 1 group to analyze body composition and physical outcomes is difficult. Therefore, we considered a sex-specific study design to reduce such biases caused by sex differences in the analysis of RET outcomes. Moreover, older female patients have significant lower muscle mass^[27]^ and strength^[28]^ than their male peers. Based on the aforementioned reasons, we conducted the present study with a sex-specific design for the older female population.

In this study, the criteria of sarcopenia diagnosis were based on the consensus of the European Working Group on Sarcopenia in Older People,^[26]^ and a method reported by Janssen was adopted, in which low relative skeletal muscle mass (ie, class I sarcopenia) in older people was associated with functional impairment and physical disability.^[29]^ According to the criteria reported by the European Working Group on Sarcopenia in Older People, the patients who only met the criteria of low muscle mass were classified as presarcopenia; sarcopenia were screened to identify those with low muscle strength and/or low physical performance adding to criteria of presarcopenia. All of the patients who were defined as presarcopenia or sarcopenia were included in the present study.

We firstly performed an outpatient screen to identify potential sarcopenia by assessing the total skeletal muscle mass (TSM), which is measured through an 8-polar bioelectrical impedance analysis device using multifrequency current (Inbody 220, Biospace, Seoul, Republic of Korea). The device can be quickly and conveniently used to measure patients’ body composition, and it has been identified to be a valid TSM estimator.^[30]^ TSM was converted into the skeletal muscle mass index (SMI) by dividing it by height in meters squared (kg/m^2^). Class I sarcopenia was defined as an SMI of 1 or more standard deviations (SDs) below the normal sex-specific means derived for young people.^[29]^ Because of the lack of an SMI standard for young adults in the local population, we used the reference value of 7.88 (0.73) kg/m^2^ for young women, which was proposed by Chien et al.^[31]^ The cutoff value of the SMI for class I sarcopenia used in the present study was less than 7.15 kg/m^2^. Furthermore, percentage body fat (BF%) was measured using bioelectrical impedance analysis and was further used to determine obesity in this study. Patients with a BF% of more than 30% were considered obese.^[32]^

### 2.3 Sample size

The sample size in this study was estimated using G∗Power 3.^[33]^ The effect size of an elastic exercise training regimen was estimated at approximately 1.08 for a timed up-and-go (TUG) task.^[34]^ At a statistical power of 0.80, an effect size of 1.08, and an alpha value of 0.05, we determined that a minimum of 40 patients would be required to identify a between-group difference of 1.3 seconds, assuming an SD of 1.19 for the TUG task. We included 46 patients according to an anticipated dropout rate of 15%.

### 2.4 Randomization

We conducted randomization after obtaining informed consent from all the patients. The patients were randomized through block allocation with a block size of 4.^[35]^ The list was computer-generated by an independent randomization center. The principal investigator informed the patients about the treatment schedules, as planned by the independent randomization center.

### 2.5 Elastic resistance exercise

Progressive RET was performed using Theraband (Hygenic Co., Akron, OH) products; the band colors, namely yellow, red, green, blue, black, and silver, denote the degree of elasticity and indicate the corresponding resistance level, with a force production of 1.32, 1.77, 2.27, 3.22, 4.40, and 5.99 kg at 100% elongation, respectively.^[36]^ Within 12 weeks of RET intervention, all patients in the EG attended 3 training sessions weekly and were supervised by a licensed senior physical therapist who was blinded to the study group assignment. Each exercise session involved a general warm-up of 10 minutes, followed by resistance training exercises (35–40 minutes), and finally a cool-down routine. The 15-point Borg scale was used to rate the patients’ perceived exertion ranging from 6 (“no exertion at all”) to 20 (“maximal exertion”) during the training sessions; this scale is a favorable tool for describing the variation in subjective intensity with physical intensity and further facilitates the estimation of the intensity of individual rehabilitation protocols.^[16]^ In resistance training, exercise loads for individual elasticity levels (as indicated by the band color) were set at a level that was perceived as slightly difficult by the patients, consequently implying a 13-grade rating on the rated perceived exertion (RPE) scale; this rating represents a moderate intensity exercise, according to the American College of Sports Medicine.^[37]^ Exercise movements were designed on the basis of previously established elastic exercise regimes used to train elderly women^[34,38]^; the movements were aimed at strengthening the main muscle groups in the trunk and the upper and lower extremities that are crucial for physical mobility.^[38]^ For each exercise movement, 3 sets involving 10 repetitions of gentle concentric and eccentric contractions through the full range of motion were slowly performed with the initial use of a yellow elastic band. The exercise intensity was increased when the patients could yield their perceived exertion on the RPE scale. If a successive advancement in the level of exercise load could not be adapted (ie, red progressing to green), the previously used elastic band color was maintained, with an additional set added to every exercise motion until the patients yielded the required effort. Details of the exercise regime and exercise progression protocol are reported in Supplemental Digital Content 1, which demonstrates the elastic resistance exercise regime, and in Supplemental Digital Content 2, which demonstrates the exercise progression protocol, respectively.

### 2.6 Outcome measures

#### 2.6.1 Body composition assessment

Body composition was measured using a Hologic QDR-1000/W whole-body dual-energy X-ray absorptiometer (Hologic, Waltham, MA). All scans and analyses were conducted by the same investigator who was blinded to the patient group assignment. We estimated the following measures: fat-free mass, leg lean mass, absolute total fat mass, and BF%.

#### 2.6.2 Measurement of muscular strength

Handgrip (HG) strength was measured using a standard hydraulic hand dynamometer (Baseline Digital, Fabrication Enterprises Inc., New York City, NY). Each patient's dominant hand was tested. The patients were seated with the arms adducted, the forearm to be tested unsupported, elbow flexed at 90°, and wrist in a neutral position. The width of the dynamometer handle was adjusted, ensuring that the middle phalange of the 3rd digit was comfortably perpendicular to the long axis of the handle. All patients were asked to perform maximal contraction by squeezing the dynamometer handle as forcefully as possible for 3 to 5 seconds, with verbal cues being provided to them for encouragement. Three trials were performed with an approximately 30-s rest between trials. The force output, measured in kilograms, was recorded for each trial, and the average of the 3 trials was considered the representative HG strength value.^[39]^

The maximal isometric strength of the quadriceps in the dominant leg was assessed using a handheld dynamometer (Microfet3; Hoggan Health Industries Co., UT). The patients were tested in the seated position, with the leg to be tested in a 45° knee flexion, and the dynamometer pad was placed immediately proximal to the lateral malleolus. All patients were asked to make their greatest effort to extend the leg against the dynamometer for 10 seconds. Three trials were performed with an approximately 30-s rest between trials.^[40]^ The maximal force output (N) was recorded for each trial, and the average of the strength levels derived from the 3 trials was considered the representative knee extensor strength.

In older adults, conducting an isometric strength test by using a handheld dynamometer has been reported to provide a high reliability level, with intraclass correlation coefficients (ICCs) of 0.97 to 0.98 for handgrip^[41]^ and 0.81 (95% confidence interval [CI]: 0.68–0.93) for knee extensor tests.^[40]^

#### 2.6.3 Muscle quality

MQ, defined as a ratio of muscular strength to muscle mass, is an indicator of muscle function.^[18]^ The MQ of the upper extremity was calculated by dividing the corresponding handgrip strength (kg) by the arm lean mass (kg). The MQ of the lower extremity was calculated by dividing the corresponding quadriceps strength (N) by the leg lean mass (kg).

#### 2.6.4 Physical capacity

Physical capacity was assessed by measuring functional mobility tasks including single-leg stance (SLS),^[42]^ gait speed,^[42]^ TUG,^[43]^ and timed chair rise (TCR) tasks.^[44]^

The SLS test, which was used to assess balance control in patients, demonstrated an acceptable reliability and validity level (ICC = 0.91, 95% CI: 0.78–0.97) for assessing balance performance.^[42]^ The SLS score represents the total time a patient can stand on 1 leg. The dominant leg of each patient was tested with the patient's eyes opened. During the SLS test, patients were asked to be barefoot with their arms placed along the sides of their bodies; they were then instructed to lift the foot not under test off the ground and flex the knee to approximately 90° while maintaining balance on the leg under test. Gait speed demonstrated acceptable reliability and validity (ICC = 0.85, 95% CI: 0.63–0.94) for measuring the time required for a patient to walk 10 minutes on a track at a self-determined pace.^[42]^ The TUG task demonstrated an acceptable reliability level (ICC = 0.98) for assessing mobility.^[43]^ The TUG task measured the time required for a patient to rise from a chair (height, 42 cm; depth, 26 cm), walk 3 m, turn around, and return to the seated position in the chair at a self-determined speed. A walking aid was used by patients during the test if necessary. We used the TCR test as a clinical measure of functional lower-extremity muscle strength in our patients. In this test, the patients stood upright from the seated position in a chair (height, 42 cm) with their arms folded across their chest and then returned to the seated position as many times as possible within 30 seconds. The TCR test demonstrated acceptable reliability (ICC = 0.97, 95% CI: 0.95–0.98) and validity (*r* = 0.89) levels for assessing the strength of the quadriceps femoris muscle group.^[44]^

### 2.7 Statistical analysis

Independent *t* tests and chi-square analyses were conducted to compare the characteristics, including age, height, weight, body mass index, prevalent comorbidities, and CIRS scores, as well as outcome measures, of the EG with those of the CG at the baseline. The Kolmogorov–Smirnov test was performed to confirm the normal distribution of all variables. An intention-to-treat analysis based on the last-observation-carried-forward technique was used to impute any missing data and to minimize bias related to loss to follow-up data. One-way analysis of covariance was performed to assess between-group differences in the posttest scores by using the baseline results, patient's age, and comorbidity scores of both groups as the covariates; moreover, the Bonferroni method for pairwise comparison tests was used to perform the post hoc analysis. The Pearson product–moment correlation coefficient (*r*) was used to assess the linear relationship between changes in body composition and physical outcomes (ie, MQ and physical capacity) at posttest. All results of comparisons with *P* < .05 were considered to be statistically significant and are presented as the mean with SD. SPSS version 17.0 was used for all analyses.

At the baseline and posttest, the patients were classified as having low muscle mass, low strength, or physical mobility difficulty on the basis of the following established cutoff values of outcome measures^[25,26]^: −2.02 kg, representing the cutoff point of the lower 20% of the distribution of residuals for appendicular lean mass, which was determined using the residual method^[45]^; <14.3 kg for handgrip^[25,26]^; <1.0 m/sec for gait speed^[25,26]^; >10 seconds for the TUG task^[46]^; <12 repetitions for the TCR test^[47]^; and <10 seconds for the SLS task.^[48]^ Patients who fulfilled 3 or more difficulties during the 5 physical tasks, namely handgrip, 10-m walk, TUG, TCR, and SLS, were defined as having physical difficulty; in addition, those who experienced no difficulty during the 5 physical tasks were defined as having nonmobility difficulty. The numbers of patients with low muscle mass, physical mobility, and nonmobility difficulty after RET in each group were calculated and analyzed using the chi-square statistic to examine differences in qualitative data between the 2 groups.

## 3 Results

Figure 1 presents the CONSORT flow diagram of patient selection and allocation in the present study. We recruited 51 eligible patients who were defined as having sarcopenic obesity. After excluding 5 patients, we finally included 46 patients in the present trial. All included patients provided informed consent, and they were subsequently randomized into the EG (n = 25) or CG (n = 21). At the baseline, all included patients were defined as obese and as having class I sarcopenia with a mean (SD) body mass index, BF%, and SMI of 27.72 (3.29) kg/m^2^, 42.89% (6.96%), and 6.89 (0.31) kg/m^2^, respectively (Table 1). Finally, 41 patients completed posttest assessments: 23 from the EG and 18 from the CG (Fig. 1). The compliance rate of the patients who participated in the exercise interventions (ie, the EG) was 97.6% without any reported side effects after elastic RET. The mean (SD) values of the patient characteristics, including age, BMI, BF%, SMI, prevalent comorbidities, and CIRS score, are presented in Table 1. No significant difference in the characteristics was observed between the patient groups. Most patients (more than 85% in each group) were considered as sedentary based on self-reported participation in a recreational physical or leisure activity (walking, running, bicycling, gardening, etc.) regularly (≥1 h/wk) within the recent 3 months.

**Table 1:**
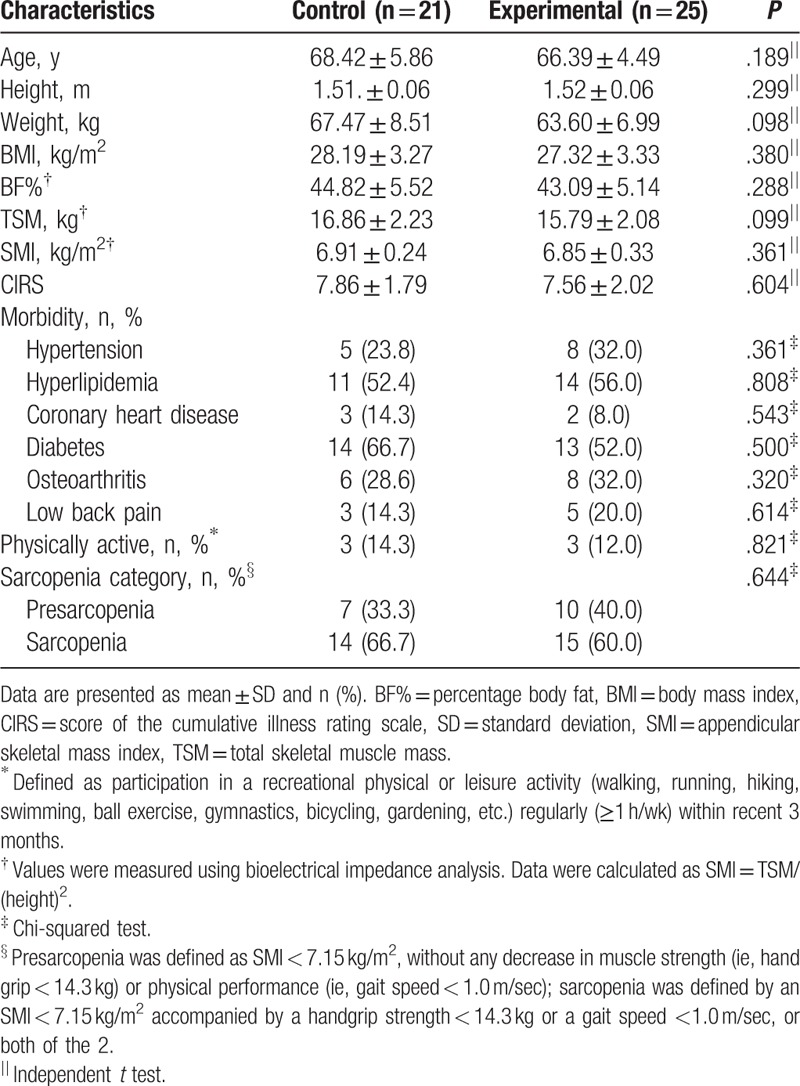
Demographic characteristics of the patients.

### 3.1 Body composition outcomes

Table 2 presents the adjusted mean change in body composition outcomes at the posttest relative to the baseline. After adjustment for baseline data, the EG exhibited significantly greater changes in fat-free mass (adjusted mean difference [aMD]: 0.73 kg; 95% CI: 0.08, 1.39; *P* < .05), leg lean mass (aMD: 0.79 kg; 95% CI: 0.45, 1.14; *P* < .001), absolute total fat mass (aMD: −1.25 kg; 95% CI: −1.98, −0.51; *P* < .01), and percent body fat (aMD: −1.83%; 95% CI: −2.60, −1.06; *P* < .001) than the CG.

**Table 2:**
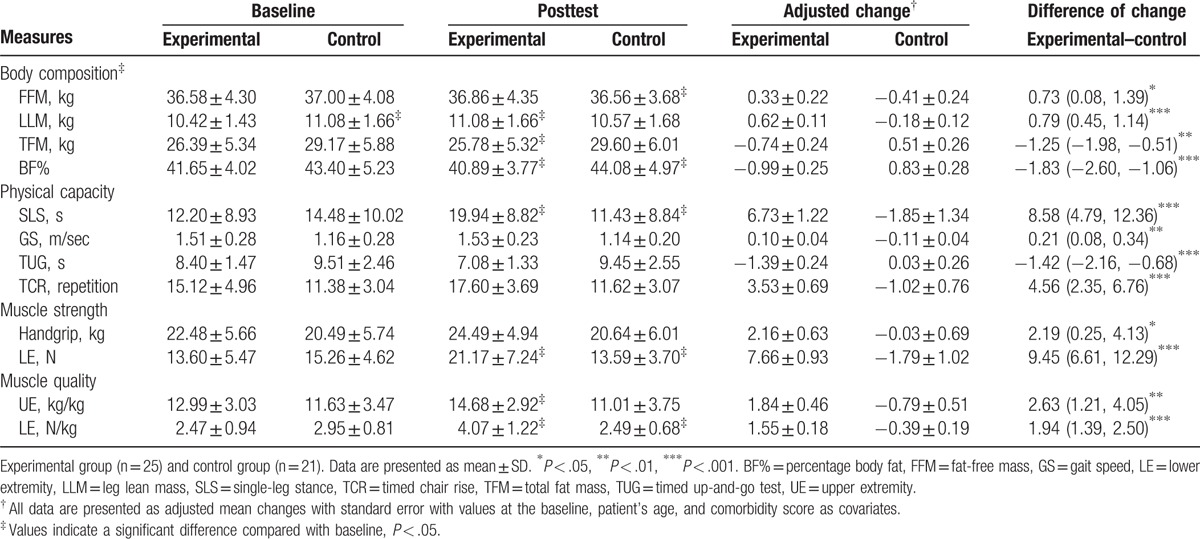
Adjusted mean change in body composition and physical capacity outcomes at posttest from baseline.

### 3.2 Physical capacity outcomes

We observed significant between-group differences in all functional mobility tasks, strength gain, and MQ at the posttest (Table 2). At the posttest, the EG exhibited significantly greater improvements in gait speed, with an aMD of 0.21 m/sec (95% CI: 0.08, 0.34; *P* < .01) than the CG. In addition, compared with the CG, the EG was 1.42 seconds (95% CI: 0.68, 2.16; *P* < .001) faster in the TUG task, performed 4.56 more repetitions (95% CI: 2.35, 6.76; *P* < .001) in the TCR test, and balanced on the leg under test longer in the SLS test (aMD 8.58 seconds; 95% CI: 4.79, 12.36; *P* < .001) at the posttest.

### 3.3 Relationship between changes in body composition and physical outcomes

Table 3 presents the Pearson correlation test results. A significant correlation was observed between leg lean mass and the MQ of the upper extremity (*r* = 0.48; *P* < .01), as well as the MQ of the lower extremity (*r* = 0.45; *P* < .01), gait speed (*r* = 0.36; *P* < .05), TUG (*r* = −0.37; *P* < .05), and TCR (*r* = 0.42; *P* < .01). Similar results were observed in the relationship of absolute total fat mass and BF% with MQ and physical capacity (Table 3).

**Table 3:**
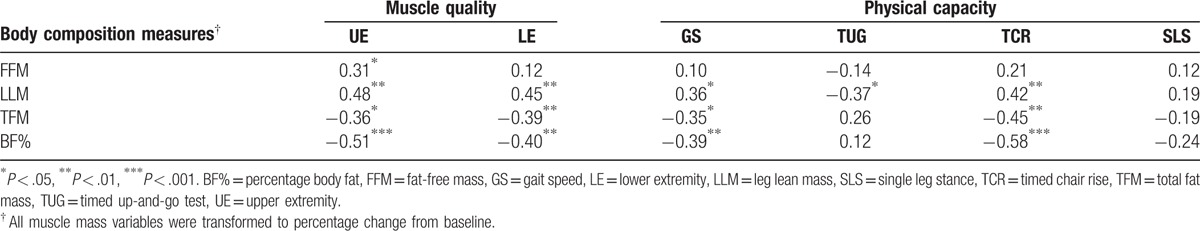
Relationship between changes in body composition and physical outcomes at posttest.

### 3.4 Effects on low muscle mass and physical difficulty

The chi-square test results revealed that elastic RET exerted effects on the prevention of low muscle mass and mobility difficulty in the EG (Table 4). After elastic RET, the EG had significantly fewer patients satisfying the criteria of low muscle mass (2 out of 25 patients; *P* = .04) and physical difficulty (2 out of 25 patients; *P* < .001) than the CG (9 and 12 out of 21 patients, respectively). Moreover, compared with the CG, the EG exhibited a significantly higher number of patients experiencing no physical difficulty (*P* = .001).

**Table 4:**
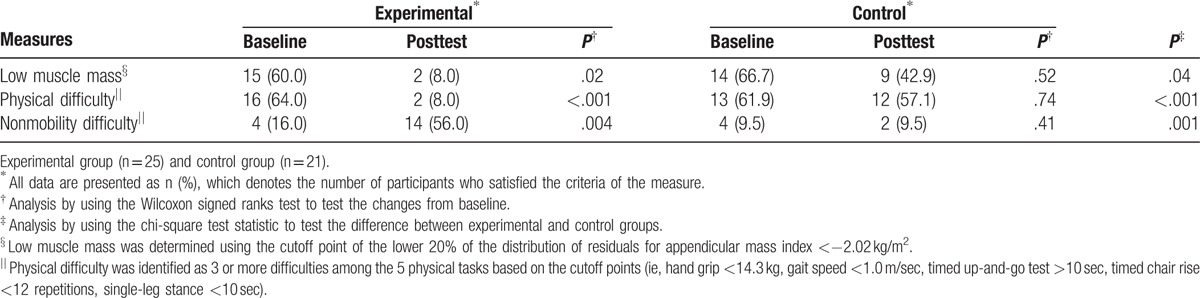
Effects of elastic resistance exercise training on muscle mass and mobility difficulty.

## 4 Discussion

In this study, we investigated the effects of elastic RET by using Theraband products for 12 weeks on body composition and physical capacity outcomes in patients with sarcopenic obesity. Compared with the CG, the EG exhibited a significant improvement in body composition (ie, increased fat-free mass and muscle indices, as well as decreased absolute total fat mass and BF%) and physical performance including the time taken for the TUG and SLS tasks, effort in the TCR task, and gait speed.

Sarcopenic obesity is a condition involving the simultaneous presence of low muscle mass and high body fat. Studies have established several methods for identifying low muscle mass, also termed class I and class II sarcopenia.^[25,26]^ The most prominent method involves setting low muscle mass at 2 SDs below the mean appendicular lean mass adjusted for body size (ie, height square), termed as appendicular mass index (kg/m^2^), in young reference groups. However, the appendicular-mass-index method might misclassify sarcopenia in an obese population.^[45]^ Studies have also suggested that body size and fat mass should be considered for identifying sarcopenia,^[45]^ particularly for estimating the prevalence of sarcopenia in overweight or obese individuals.^[45]^ In the present study, we used the cutoff value of the residual appendicular lean mass adjusted for height and fat mass to identify low muscle mass in obese patients who were undergoing elastic RET; after 12 weeks of elastic RET, the EG had significantly fewer patients with low muscle mass than the CG, with 8.0% of the EG and 42.9% of the CG considered to have sarcopenia.

The present study also demonstrated that changes in body composition were significantly correlated with muscle strength and physical mobility outcome after RET intervention. Considering that muscle mass loss is associated with aging and that RET is advantageous for muscle mass and strength gain in elderly individuals,^[49–51]^ RET combined with or without other exercise types may enable obese elderly individuals to overcome muscle-attenuated physical difficulties.^[49]^ RET has been reported to be beneficial for physical function by increasing muscle protein synthesis and muscle strength in frail elderly individuals with sarcopenia or in those with obesity.^[2,13]^ Because loss of muscle mass, particularly in the lower limb muscle groups, leads to further mobility difficulty and functional limitations in elderly individuals,^[3,4,7]^ our findings of a simultaneous increase in leg lean mass and improved physical mobility levels after RET may be explained by the association between low muscle mass and the high risk of mobility limitations. In addition, obese elderly individuals with low muscle mass have a higher risk of mobility limitation or physical difficulty than those with sarcopenia or obesity alone.^[6–9]^ Therefore, RET is suggested for elderly individuals with sarcopenic obesity. Our study results indicate that RET not only improves the body composition in obese elderly individuals but also facilitates the increase in muscle mass, which may further benefit physical function by improving muscle strength.

Elastic band RET has been recently used for elderly individuals who are obese^[20,21,38]^ or not obese.^[14,19,34,52]^ Overall, these studies have used exercise protocols with an intervention period of 8 to 24 weeks, a frequency of 2 to 5 times per week, and a low to moderate exercise intensity level. In general, studies have reported significant improvements in body composition, as indicated by significantly decreased fat mass and increased lean mass,^[20,38]^ as well as muscle structural changes,^[14]^ strength gain,^[14,20,38]^ and functional mobility.^[19,21,34,38]^ Our findings in elderly patients with sarcopenia or obesity are in concordance with those of previous studies on elastic resistance exercise in older adults.

The present study has certain limitations. First, the study included only female patients. Because of the sex-specific response to RET, our results might not be generalizable to all elderly populations. Moreover, the patients in this study were young, which could represent a bias for elderly female adults. Some of the included patients had a BMI of <27 kg/m^2^, which is the cutoff value of obesity for the Asian population.^[53]^ However, BMI indicates only changes in total body mass, and it may mask the changes in a person's body composition; hence, BF% can be used to define obesity rather than BMI, to analyze the treatment effect on body composition changes. Therefore, we considered older women with a BF% higher than 38% as obese.^[32]^ Second, the small sample size limited the identification of the association between improved body composition and physical mobility levels, despite low muscle mass and high body fat being identified to be associated with lower physical function and higher mobility limitation.^[3,5]^ Third, we did not assess physical activity levels by using self-reported questionnaires such as the International Physical Activity Questionnaire or by using measuring devices such as accelerometers. Finally, we did not analyze a diet or nutrition-supplement control during the intervention. We could not draw conclusions about the association between nutrition supplementation and changes in body composition during RET. Diet patterns or nutrition supplements such as protein supplements may interfere with changes in the whole body weight or muscle mass during RET.^[39]^

## 5 Conclusion

This prospective study revealed that 12 weeks of elastic RET exerted positive effects on the body composition and functional mobility outcomes in elderly women with sarcopenic obesity. The study results suggest that greater emphasis should be placed on elastic RET for enabling patients with sarcopenic obesity, particularly obese older women with class I sarcopenia, to gain muscle mass and strength. The elastic RET protocol and the study findings could facilitate clinical decision-making regarding the optimal treatment strategy for obese elderly women, particularly for those with class I sarcopenia.

## Supplementary Material

Supplemental Digital Content
