# Supplementary material for: Effects of elastic resistance exercise on body composition and physical capacity in older women with sarcopenic obesity: A CONSORT-compliant prospective randomized controlled trial
Source: Medicine (Baltimore). 2017 Jun 8;96(23):e7115. doi: 10.1097/MD.0000000000007115 (PMC5466239; doi:10.1097/MD.0000000000007115)
Supplement: Supplemental Digital Content [file medi-96-e7115-s001.docx]

Supplemental Digital Content 1

Supplemental Digital Content 1. Table that demonstrates the details of the elastic resistance exercise regime.

**Elastic resistance exercise regime**

| Movement | Intensity  (repetition/set) | Targeted muscle group | Duration  (min) |
| --- | --- | --- | --- |
| A. Warm-up |  |  |  |
| 1. Mobility exercise of the neck, upper limbs, and back |  | Upper quarter flexors and extensors | 5 |
| 2. Global flexion–extension of the lower limb |  | Lower quarter flexors and extensors | 5 |
| B. Upper quarter |  |  |  |
| 1. Seated chest press | 10–20/3 | Upper quarter extensors | 5–10 |
| 2. Seated row | 10–20/3 | Upper quarter flexors | 5–10 |
| 3. Seated shoulder press | 10–20/3 | Shoulder gargle muscle groups | 5–10 |
| C. Lower quarter |  |  |  |
| 1. Concentric–eccentric hip circumduction | 10–20/3 | Hip gargle muscle groups | 5–10 |
| 2. Leg press | 10–20/3 | Lower quarter extensors | 5–10 |
| 3. Leg curl | 10–20/3 | Lower quarter flexors | 5–10 |
| D. Cool down |  |  | 5 |

Supplemental Digital Content 2. Table that demonstrates the details of the progress of elastic resistance training protocol.

Supplemental Digital Content 2

**Exercise progression protocol**

|  | Week | 1 | 2 | 3 | 4 | 5 | 6 | 7 | 8 | 9 | 10 | 11 | 12 |
| --- | --- | --- | --- | --- | --- | --- | --- | --- | --- | --- | --- | --- | --- |
| Theraband color | Yellow | X | X |  |  |  |  |  |  |  |  |  |  |
|  | Red |  |  | X | X |  |  |  |  |  |  |  |  |
|  | Green |  |  |  |  | X | X |  |  |  |  |  |  |
|  | Blue |  |  |  |  |  |  | X | X |  |  |  |  |
|  | Black |  |  |  |  |  |  |  |  | X | X |  |  |
|  | Silver |  |  |  |  |  |  |  |  |  |  | X | X |
| Exercise loading | Repetition | 10–15 | 15–20 | 10–15 | 15–20 | 10–15 | 15–20 | 10–15 | 15–20 | 10–15 | 15–20 | 10–15 | 15–20 |
|  | Set | 3 | 3 | 3 | 3 | 3 | 3 | 3 | 3 | 3 | 3 | 3 | 3 |
|  | RPE^a^ | 10–13 | 10–13 | 10–13 | 10–13 | 10–13 | 10–13 | 10–13 | 10–13 | 10–13 | 10–13 | 10–13 | 10–13 |

X = color of the Theraband used

^a^Ratings of perceived exertion by using the Borg scale
